# Effectiveness of Physiotherapy Interventions on Executive Function in Patients with Chronic Pain: A Systematic Review

**DOI:** 10.3390/neurolint18030055

**Published:** 2026-03-16

**Authors:** Aser Donado-Bermejo, Silvia Di-Bonaventura, Pablo Barrenechea-Leal, Francisco Mercado-Romero, Marisa Fernández-Sánchez, Raúl Ferrer-Peña

**Affiliations:** 1 Escuela Internacional de Doctorado, Rey Juan Carlos University, 28008 Madrid, Spain; a.donado.2023@alumnos.urjc.es (A.D.-B.); pablo.020@hotmail.com (P.B.-L.); 2 Cognitive Neuroscience, Pain and Rehabilitation Research Group (NECODOR), Faculty of Health Sciences, Rey Juan Carlos University, 28922 Madrid, Spainmarialuisa.fernandez@urjc.es (M.F.-S.); 3 Faculty of Health Sciences, UNIE University, 28029 Madrid, Spain; 4 PainCorp Foundation, Madrid, Spain; 5 Department of Psychology, Faculty of Health Sciences, Universidad Rey Juan Carlos, 28922 Madrid, Spain; 6 Clinico-Educational Research Group on Rehabilitation Sciences (INDOCLIN), Centro Superior de Estudios Universitarios La Salle, Universidad Autónoma de Madrid, 28023 Madrid, Spain; 7 Centro de Salud Entrevías, Gerencia Asistencial de Atención Primaria de la Comunidad de Madrid, 28023 Madrid, Spain

**Keywords:** chronic pain, executive function, neuromodulation, physiotherapy, exercise therapy, cognitive dysfunction

## Abstract

**Background:** Chronic pain is a prevalent and disabling condition that affects physical health but also cognitive domains. Executive functions, including inhibitory control, cognitive flexibility, and working memory, essentials for self-regulation, treatment adherence, and coping with symptoms, are particularly compromised. Physiotherapy interventions, traditionally aimed at physical outcomes, may also influence executive functions; however, their impact remains unclear. **Objective:** This review aimed to synthesize current evidence regarding the effects of physiotherapy-related interventions on executive function in adults with chronic pain. **Methods:** The review followed the Cochrane Handbook and Preferred Reporting Items for Systematic Reviews (PRISMA) guidelines, and the protocol was registered in PROSPERO (CRD42024611800). A comprehensive search was performed. Randomized controlled trials (RCTs) included adults with chronic pain (≥3 months) whose executive function outcomes were evaluated after physiotherapy-based interventions. **Results:** Out of 12,391 records, 10 randomized controlled trials were included. Populations primarily had fibromyalgia, chronic low back pain, and chronic musculoskeletal pain. Interventions encompassed transcranial direct current stimulation (tDCS), transcranial magnetic stimulation (rTMS), neurofeedback, structured exercise, and multimodal physical-cognitive-mindfulness training. Intervention durations ranged from one session to 16 weeks. Executive function was assessed with diverse neuropsychological tests. tDCS improved attention, inhibitory control, cognitive flexibility, and working memory. Exercise interventions showed benefits in working memory and inhibitory control. **Conclusions:** Preliminary evidence suggests that physiotherapy interventions, particularly anodal tDCS and structured exercise, may improve executive functions in individuals with chronic pain. Future trials should incorporate long-term follow-up. Integrating cognitive targets into physiotherapy may enhance the multidimensional management of chronic pain.

## 1. Introduction

Chronic pain, defined as pain persisting or recurring for over three months [1], is a debilitating condition with a large societal impact [2], affecting approximately 20% of the global adult population [3] and posing a substantial burden on healthcare systems [4].

Beyond sensory and emotional dimensions, chronic pain involves significant cognitive sequelae [5,6,7,8]. Emerging evidence indicates that patients experience impairments in higher-order functions, adversely impacting daily living and treatment adherence [9,10]. Specifically, domains such as attention, memory and executive functioning are negatively affected [7,11], likely due to overlapping neural mechanisms in pain processing and cognitive regulation [8,12,13,14].

Cognitive dysfunction may impair effective self-management, potentially perpetuating the pain experience [7]. Among affected domains, executive functions (EFs) are particularly disrupted [15,16].

EFs encompass interrelated processes enabling goal-directed behavior, including inhibitory control, cognitive flexibility, and working memory [17], alongside complex functions like planning and reasoning [18]. Together, these processes provide the cognitive basis for managing complex tasks, making adaptive decisions, and exerting behavioral self-regulation.

When compromised, these abilities can hinder an individual’s capacity to engage in effective pain management and adherence to treatment plans [16,19], ultimately contributing to chronification and intensification of pain experience [7] and also to everyday interference of pain [19] on cognitive and physical functioning.

Given that EFs influence behavioral regulation, interventions targeting both physical and cognitive dimensions are valuable. Physiotherapy offers such an avenue. Beyond physical benefits, interventions like exercise and education [20,21,22] inherently require patients to plan, regulate effort, sustain attention, and adapt to changing demand, and may also influence cognitive functioning. Combined with biological mechanisms like neuroplasticity and reduced inflammation [23,24,25], physiotherapy may plausibly modulate EFs.

Nevertheless, the extent to which physiotherapy interventions specifically influence EFs in chronic pain populations remains unclear.

While a growing body of literature addresses cognitive impairments in chronic pain populations [6,8,14,26,27], evidence regarding the impact of physiotherapy-based interventions on EFs is scattered and heterogeneous. For instance, a systematic review by Higgins et al. [27] reported consistent negative associations between chronic pain and domains, such as attention, memory, processing speed, and executive function, highlighting that executive processes are among the most vulnerable cognitive domains in this population. Previous reviews have primarily examined the cognitive consequences of chronic pain itself [13,28] or have addressed the broader cognitive impact of physiotherapy and rehabilitation interventions in other populations [29,30,31].

Moreover, no previous synthesis has systematically evaluated randomized controlled trials (RCTs) to determine whether physiotherapy-related interventions exert a measurable effect on executive function outcomes in adults with chronic pain. This represents a relevant gap, as the absence of consolidated evidence limits the integration of executive function endpoints into physiotherapy-based pain management strategies.

This systematic review aimed to answer the following question: In adults with chronic pain lasting at least three months, do physiotherapy-related interventions, compared to any type of comparator, lead to improvements in EFs, as measured by validated assessments, in RCTs?

For this review, “physiotherapy-related interventions” were operationally defined as interventions delivered within the scope of physiotherapy practice, including structured therapeutic exercise, movement-based therapies, physical activity programs, patient education, or multimodal interventions in which physiotherapy constituted a core component. This definition was grounded in the use of non-ionizing physical agents and therapeutic movement strategies commonly employed within physiotherapy, encompassing both conventional approaches (e.g., exercise-based interventions and education) and neuromodulatory techniques based on physical energy delivery, such as rTMS and tDCS, when applied within a rehabilitation framework [32].

By addressing this gap, we aim to inform clinical practice and guide future research directions toward a more comprehensive management of chronic pain that incorporates executive function outcomes as core components of assessment and rehabilitation.

## 2. Materials and Methods

This systematic review was conducted following the guidelines of the Cochrane Handbook for Systematic Reviews of Interventions [33] and the most recent guide, PRISMA (Supplementary File S1) [34]. The protocol was registered in PROSPERO with ID CRD42024611800 in December 2024.

### 2.1. Search Strategy

A systematic search for studies was conducted in December 2024, actualized in July 2025, across PubMed, Scopus, Embase, CINAHL, and PsycInfo. The strategy combined Medical Subject Headings (MeSH) and free-text keywords relating to three main concepts: (i) chronic pain conditions (e.g., “Chronic Pain,” “Chronic Musculoskeletal Pain”), (ii) physiotherapy-related interventions (e.g., “Physical Therapy Modalities,” “Exercise Therapy”), and (iii) executive functions (e.g., “Executive Function,” “Working Memory,” “Cognitive Flexibility”).

The complete search strings, adapted to each database, are provided in Appendix A to ensure reproducibility. No searches of clinical trial registries or other grey literature sources were conducted. Grey literature was not included because the review was restricted to peer-reviewed randomized controlled trials to ensure methodological consistency and quality appraisal using validated tools, which require full methodological reporting. As such, the potential influence of unpublished or ongoing trials could not be assessed. Due to the anticipated clinical and methodological heterogeneity across interventions, populations, and outcome measures, and because a meta-analysis was not planned, formal assessment of publication bias (e.g., funnel plot analysis) was not applicable. 

### 2.2. Selection Criteria

#### 2.2.1. Types of Studies

Only original studies of human clinical trials published without language restrictions were selected. Letters, posters or communications, abstracts, editorials, reviews, case series, and single-arm studies were excluded. No time restrictions were placed.

Studies had to be RCTs. The selection criteria used in the present systematic review were based on clinical and methodological factors, such as the PICO-S described by Stone et al. [35].

#### 2.2.2. Population

Inclusion criteria were as follows:

- Adults (≥18 years) with chronic pain persisting for at least three months.
- Studies evaluating EFs.
- Interventions primarily based on physiotherapy or analogous therapeutic modalities.

Exclusion criteria were as follows:

- Pain of less than three months’ duration.
- Pain associated with active oncological processes or ongoing cancer treatment.
- Participants with cognitive impairments attributable to unrelated neurodegenerative diseases, psychiatric disorders, or other comorbidities known to independently affect cognition.
- Pediatric populations (<18 years).
- Studies focusing exclusively on pharmacological or surgical interventions without an integrated physiotherapy component.

#### 2.2.3. Intervention

Eligible interventions include physiotherapy-related approaches, such as therapeutic exercise, patient education, manual therapy, or multidisciplinary rehabilitation programs where physiotherapy constitutes a central element. For eligibility, interventions were defined as non-pharmacological therapeutic approaches based on the application of physical agents or movement-based strategies within the scope of physiotherapy practice.

#### 2.2.4. Comparator

Comparators will include usual care, placebo or sham interventions, no intervention, alternative non-pharmacological therapies, or multidisciplinary programs where physiotherapy is not the primary intervention.

#### 2.2.5. Outcome Measures

The primary outcome is the effect of physiotherapy interventions on EFs in adults with chronic pain. Executive function is conceptualized as a set of higher-order cognitive processes essential for goal-directed behavior [17,18], and will be operationalized in this review through the following domains commonly assessed in research:

- Working memory,
- Processing speed,
- Problem-solving,
- Decision-making,
- Cognitive flexibility,
- Inhibitory control.

Quantitative outcomes derived from standardized neuropsychological assessments or other validated instruments specifically designed to evaluate executive function domains will be included.

### 2.3. Study Selection

**Figure 1:**
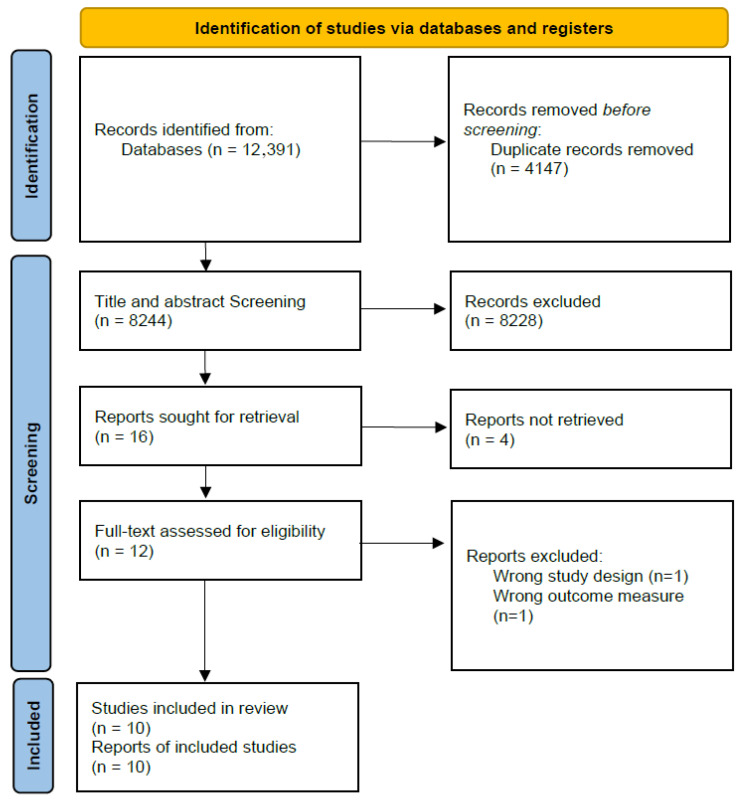
PRISMA 2020 flow diagram of study selection. The diagram illustrates the number of records identified, screened, excluded, and included at each stage of the systematic review process, following the PRISMA 2020 guidelines.

After addressing potential duplicates, two reviewers independently screened titles and abstracts based on the predefined inclusion and exclusion criteria. The screening process was conducted using Rayyan QCRI (https://www.rayyan.ai/) [36]. Full-text articles of potentially eligible studies were then retrieved and assessed for final inclusion. Any disagreements between reviewers were resolved through discussion or, when necessary, by consulting a third reviewer. The entire selection process was documented using a PRISMA flow diagram (Figure 1).

### 2.4. Data Extraction

Data were extracted independently by two reviewers using a standardized data extraction form. Extracted data included study characteristics, participant demographics, details of the intervention and comparator, outcome measures related to executive function, and main findings. Discrepancies in data extraction were resolved by consensus or by consulting a third reviewer.

### 2.5. Methodological Quality Assessment

The methodological quality and risk of bias of the included RCTs were assessed independently by two reviewers using the Revised Cochrane Risk of Bias Tool for Randomized Trials (RoB 2) [37] and Physiotherapy Evidence Database (PEDro) scale [38].

Two reviewers independently performed the quality assessment, and disagreements were resolved through consensus or consultation with a third reviewer when necessary. The results of the quality assessment were presented both narratively and in tabular format. Given the absence of quantitative synthesis, risk of bias findings were interpreted descriptively to contextualize the strength and consistency of the evidence rather than to weight pooled effect estimates.

**Table 1:** Characteristics of included studies and summary of EF outcomes. Sample characteristics, intervention protocols, comparator groups, cognitive domains assessed, and main findings are summarized for each study. Cognitive outcomes are grouped under EF domains and reported along with the assessment tools used. All studies included involved adult participants with chronic pain.

Study	Diagnosis and Sample Characteristics	Intervention Description and Duration	Comparator Group	Executive Domain	Test Used	Main Findings	Funding	Conflicts of Interest
Alcon C. et al. 2025 [39]	Chronic low back pain *n* = 20 (18 women)	tDCS (2 mA, anodal, left DLPFC) + PNE 5 sessions over 2 weeks	Sham tDCS + PNE	Inhibitory control; working memory	SCWT, CTMT2 Inhibitory and Set-Shifting tasks	Improvements in inhibitory control and executive function following combined tDCS and pain neuroscience education.	Yes (public/academic)	No conflicts.
Massah N. et al. 2025 [40]	Chronic low back pain *n* = 40; adults (25–40 years)	VR-based exergaming. Single session 30–45 min session	Single session of core stability exercises.	Attention and working memory; inhibitory control	N-back test; Simple Stroop Task	Exergaming led to greater improvements in working memory, processing speed, and inhibitory control compared to core stability exercises	Yes (public/academic)	No conflicts.
Vicuña Serrano P. et al. 2022 [41]	Fibromyalgia *n* = 36; women (30–65 years)	a-tDCS: 2 mA (20 min) 5 consecutive days over 4 weeks, totaling 20 sessions.	Sham tDCS	Processing speed; attention; executive functions (cognitive flexibility, set-shifting, inhibitory control); verbal fluency; working memory	TMT A-B, COWAT, Digits Subtest of WAIS-III	Home-based anodal tDCS over the left DLPFC improved executive function, semantic verbal fluency, attention, and short-term working memory, with no effects on orthographic fluency or backward working memory.	Yes (public/academic)	No conflicts.
Bilir I. et al. 2021 [42]	Fibromyalgia syndrome *n* = 20	rTMS 2 weeks (5 sessions/week) + 4 weeks (1 session/week)	Sham rTMS	Attention; memory; verbal fluency	Addenbroke’s Cognitive examination revised: attention and orientation, memory, verbal fluency, etc.	No significant improvements were observed following neuronavigated rTMS compared to sham stimulation.	Yes (public/academic)	No conflicts.
Wu Y. et al. 2021 [43]	Fibromyalgia *n* = 80; adults (60 intervention, 20 control)	Neurofeedback training 8 weeks (20 sessions, 30 min each)	Telephone support group	Sustained attention; working memory	PVT and DST	Neurofeedback improved sustained attention but did not produce significant changes in working memory or broader executive functions.	Yes (public/academic)	No conflicts.
Norouzi E. et al. 2019 [44]	Fibromyalgia *n* = 60; women	Zumba dancing program Aerobic exercise training on a walking treadmill 12 weeks	Group meeting, maintain current physical activity levels, and refrain from additional exercise or sport activities.	Working memory	N-back test	Zumba dancing and aerobic exercise were associated with improvements in working memory.	Not declared	Not declared
Souza dos Santos V. et al. 2018 [45]	Fibromyalgia *n* = 40; women (18–65 years)	a-tDCS combined with online working memory training 8 consecutive sessions (weekdays)	Sham-tDCS + WMT	Working memory; attention span; verbal fluency; verbal episodic memory	WAIS-III Digit Span (forward and backward), COWAT, RAVLT	Anodal tDCS combined with working memory training improved immediate memory, verbal fluency, and short-term working memory, with no effects on delayed memory or complex attention.	Yes (public/academic)	No conflicts.
Ferreira Silva A. et al. 2017 [46]	Fibromyalgia *n* = 20	Online combined tDCS and Go/No-go Task Single session	Sham tDCS	Executive attention and inhibitory control	Attention Network Test (ANT)	Anodal tDCS over the left DLPFC improved executive control and reduced attentional interference, with no significant phase or interaction effects.	Yes (public/academic)	No conflicts.
Jay K. et al. 2016 [47]	Chronic musculoskeletal pain *n* = 112; female laboratory technicians	Physical Cognitive Mindfulness training (PCMT) 10-week intervention	Adhering to on-going company initiatives	Executive functions (cognitive flexibility, inhibitory control, working memory); attention	CNS Vital Signs: shifting attention, continuous performance, and stroop tests	Physical–cognitive–mindfulness training led to within-group improvements in executive function, with no significant between-group differences compared to the reference intervention.	Not declared	No conflicts.
Munguía-Izquierdo D. et al. 2008 [48]	Fibromyalgia *n* = 60; women (18–60 years)	Aquatic exercise 16 weeks (3 times/week)	Instruction not to change their habits regarding physical activities during the period.	Sustained and divided attention; processing speed; interference control	Paced Auditory Serial Addition Test (PASAT)	Moderate-intensity aquatic exercise improved executive function, cognitive performance, and overall functional outcomes compared to control.	Yes (public/academic)	No conflicts.

## 3. Results

### 3.1. Literature Search

The full electronic search yielded 12,391 records. After removing duplicates, a total of 8244 records were screened. Out of these, 8228 records were excluded for clearly not meeting eligibility criteria, including irrelevant populations, inappropriate interventions, or outcomes unrelated to EFs.

This process resulted in 16 full-text articles assessed for eligibility. Four reports were not retrieved after reasonable attempts to obtain them. Among the 12 full-text articles assessed, two were excluded: one due to an inappropriate study design and one for inappropriate outcomes. Consequently, 10 RCTs were included.

The search was conducted by the first author (DBA). Screening of titles/abstracts and full texts was independently performed in duplicate by two reviewers (DBA and BLP). Discrepancies were discussed and resolved by consensus, and when necessary, referred to a third party. The selection process is detailed in the PRISMA flow diagram (Figure 1).

### 3.2. Description of Included Studies

Table 1 summarizes the characteristics of the 10 RCTs included, encompassing a range of chronic pain conditions. Seven studies (70%) involved individuals with fibromyalgia [41,42,43,44,45,46,48], two (20%) investigated chronic low back pain [39,40], and one (10%) addressed chronic musculoskeletal pain [47].

With respect to interventions, tDCS was applied in four studies (40%) [39,41,45,46], either alone or in combination with educational strategies. Two studies (20%) evaluated rTMS [42] and neurofeedback [43], respectively. Three studies (30%) implemented structured physical exercise programs, including exergames [40], Zumba [44], and aquatic exercise [48]. One trial (10%) investigated a multimodal approach combining physical, cognitive, and mindfulness training [47].

Intervention durations ranged from a single session to 16 weeks. Five studies (50%) compared the intervention with a sham intervention [39,41,42,45,46], three studies (30%) compared with active controls [40,43,47], while two trials (20%) used passive control groups [44,48].

EFs were assessed with a wide range of measures addressing distinct domains, including attentional interference [39,40], cognitive flexibility [39,41], working memory [40,41,43,44,45], verbal fluency [41,45], and global executive attention [42,46,47].

Due to substantial heterogeneity, a meta-analysis was not feasible.

### 3.3. Methodological Quality

The methodological quality of the included RCTs was assessed using the PEDro scale, with total scores ranging from five to ten. The mean PEDro score across studies was 8.3 (SD = 1.4), indicating an overall high methodological quality (Table 2).

All trials satisfied the criteria for eligibility specification, random allocation, and between-group comparisons. However, subject blinding and therapist blinding were rarely achieved. Concealed allocation was described in seven out of ten trials. Assessor blinding and adequate follow-up were generally well managed across studies. Intention-to-treat analysis was reported in only two studies.

These findings suggest that, while the internal validity and reporting quality of most trials were acceptable, the lack of subject and therapist blinding, together with the limited use of intention-to-treat analysis, remain important limitations that should be considered.

The risk of bias of the included RCTs was assessed using the RoB 2 Tool. The overall risk of bias was judged as low in five studies, some concerns in four studies, and high in one study.

Regarding specific domains, the randomization process was judged as low risk in nine trials, with one study rated as having some concerns. Deviations from intended interventions were also consistently low risk, except for one study with some concerns. For missing outcome data, nine trials were rated as low risk, and one presented some concerns. Measurement of the outcome was rated as low risk in eight studies, with two judged as having some concerns. The domain with the greatest variability was the selection of the reported result, where six studies were rated as low risk, three as having some concerns, and one as high risk.

Overall, most trials demonstrated acceptable methodological quality regarding bias control. However, the variability in selective reporting and outcome measurement, together with one trial judged at high overall risk of bias, highlights important limitations that should be considered.

**Figure 2:**
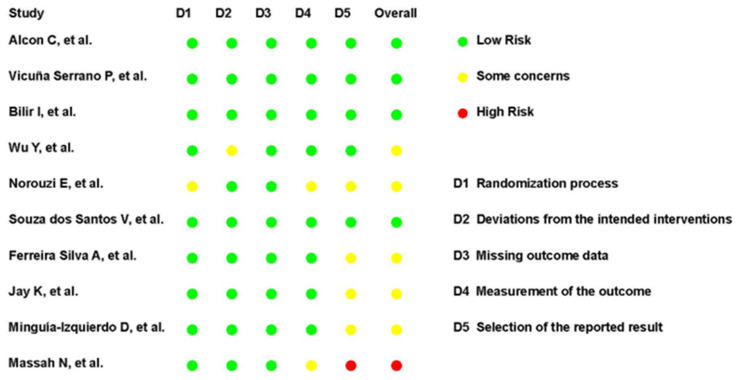
Summary of risk of bias assessment using RoB 2. Domains evaluated were D1, randomization process; D2, deviations from intended interventions; D3, missing outcome data; D4, measurement of the outcome; and D5, selection of the reported result. Traffic-light plots indicate judgments of low risk (green), some concerns (yellow), or high risk (red) [39,40,41,42,43,44,45,46,47,48].

**Figure 3:**
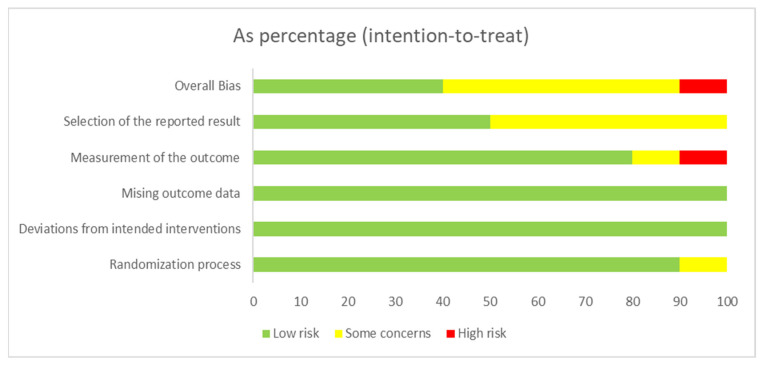
Proportional risk of bias across included RCTs, assessed with RoB 2. Results are presented as percentages of studies rated low risk (green), some concerns (yellow), or high risk (red) for each domain and overall.

The complete judgments for each study are summarized in Figure 2 and Figure 3.

Inter-rater reliability was evaluated for both methodological quality and risk of bias. For the PEDro scale, item-level agreement ranged from 80% to 100%, with kappa coefficients between 0.60 and 1.00, indicating moderate to almost perfect agreement. The intraclass correlation coefficient (ICC) for the total PEDro score demonstrated excellent reliability (ICC = 0.90). Regarding the RoB 2 Tool, agreement across domains ranged from 70% to 100%, with weighted kappa values between 0.40 and 0.85, reflecting fair to substantial agreement. Overall, these results support a high level of consistency between the two independent reviewers.

### 3.4. Effects of Physiotherapy on Executive Function

#### 3.4.1. Neuromodulation Interventions

Several trials explored the impact of non-invasive brain stimulation on EFs. One trial [39] shows that active anodal transcranial direct current stimulation (a-tDCS) over the left dorsolateral prefrontal cortex (DLPFC), combined with Pain Neuroscience Education (PNE), led to significant improvements in selective attention and inhibitory control, as assessed by the Stroop Color Word Test (SCWT) and the Comprehensive Trail Making Test (CTMT).

Similarly, another trial [41] reported that home-based a-tDCS over the left DLPFC in fibromyalgia patients significantly improved cognitive flexibility and semantic fluency, with additional benefits for working memory, but not for orthographic fluency or backward span.

Another trial [45] found that combining tDCS with working memory training enhanced immediate memory and short-term working memory in fibromyalgia patients, though no effects were observed on delayed memory or processing speed.

In contrast, a trial [46] observed that a single session of a-tDCS over the DLPFC modulated attentional networks with indirect implications for executive control.

On the other hand, a trial [42] examined the effects of neuronavigated rTMS and found no clinically meaningful improvements in global neurocognitive performance in fibromyalgia patients, despite modest between-group differences in total cognitive score and attention subscales.

#### 3.4.2. Cognitive Training and Neurofeedback

Wu et al. [43] investigated the effects of neurofeedback training targeting sensorimotor and alpha rhythms in fibromyalgia patients and observed a significant improvement in sustained attention. However, no significant changes were found in working memory performance, suggesting a selective benefit for attentional domains rather than broader EFs.

#### 3.4.3. Physical Exercise and Multimodal Interventions

Several studies evaluated the role of physical activity in modulating cognitive performance. One trial [44] reported that both Zumba and aerobic exercise significantly enhanced working memory in women with fibromyalgia. These results suggest that structured physical activity may contribute to improved executive outcomes in this population.

Another trial [48] found that a 16-week aquatic exercise program led to improvements in cognitive function, sleep, and physical function among women with fibromyalgia, with the cognitive effect measured using the Paced Auditory Serial Addition Task (PASAT).

In contrast, another trial [47] investigated a 10-week physical-cognitive-mindfulness training (PCMT) program in female workers with chronic musculoskeletal pain and found no between-group differences in neurocognitive performance or neuromuscular function. This may reflect limitations in intervention intensity, participant characteristics, or outcome sensitivity.

Finally, a trial [40] evaluated a structured aerobic exercise program in patients with fibromyalgia and reported improvements in EFs, specifically in inhibitory control and cognitive flexibility, as measured by the SCWT and the Wisconsin Card Sorting Test (WCST). These findings further support the role of aerobic training as a non-pharmacological strategy to enhance executive function in chronic pain populations.

### 3.5. Narrative Synthesis by Executive Function Domain

To provide a structured interpretation of findings across heterogeneous interventions, results were synthesized according to executive function domains, and validated neuropsychological instruments were classified based on their primary cognitive demand as presented in Table 3.

Working memory was the most frequently assessed domain. Improvements were observed primarily in studies employing tDCS combined with cognitive training [45] and structured physical exercise programs [44,48]. However, effects were not consistent across all neuromodulation trials, with some studies reporting selective short-term gains but no sustained improvements.

Inhibitory control showed positive responses, particularly in studies using anodal tDCS over the dorsolateral prefrontal cortex [39] and aerobic exercise interventions [40], as assessed by the Stroop Color Word Test. These findings suggest potential modulation of prefrontal networks involved in interference control.

Cognitive flexibility demonstrated improvements in several neuromodulation studies [39,41] and in aerobic exercise interventions [40], as measured by the WCST and Trail Making paradigms. Nevertheless, variability in intervention duration and stimulation parameters may account for inconsistent findings across trials.

Executive attention yielded mixed results. While some neuromodulation and neurofeedback studies reported enhanced attentional regulation [43,46], these effects were often domain-specific and did not consistently generalize to broader executive performance.

Processing speed was less consistently improved, with limited evidence suggesting modest gains in selected neuromodulation protocols, but no robust pattern across intervention types.

Overall, working memory and inhibitory control appear to be the executive domains most responsive to physiotherapy-related interventions, whereas evidence for improvements in cognitive flexibility, executive attention, and processing speed remains heterogeneous and intervention-dependent.

## 4. Discussion

This systematic review synthesized the current evidence regarding the effects of physiotherapy-related interventions on executive function in adults with chronic pain. Preliminary findings suggest that neuromodulation techniques, particularly a-tDCs over the DLPFC, may influence specific EFs, such as inhibitory control and working memory. However, these findings remain inconsistent and are largely derived from small and heterogeneous trials, warranting cautious interpretation [39,41,45,46] and limiting the overall certainty and generalizability. In addition, other approaches, such as rTMS and PCMT, showed even more heterogeneous or limited effects [42,48].

In relation to rTMS, although high-frequency stimulation over the left DLPFC has demonstrated level A or B evidence for analgesic effects in certain chronic pain conditions according to international guidelines [53], its impact on executive functions remains considerably less robust and less consistent. Recent meta-analyses examining DLPFC-targeted rTMS have suggested small-to-moderate effects on working memory and cognitive control in neurological populations [54], yet such benefits have not been consistently replicated in chronic pain cohorts. More specifically, randomized controlled trials in fibromyalgia and other chronic pain conditions have reported either non-significant changes in global cognitive scores or improvements that did not survive between-group comparisons when contrasted with sham stimulation [55,56]. Taken together, the current evidence indicates that while rTMS holds established therapeutic value for pain modulation, its specific and reproducible effects on executive functioning in chronic pain populations remain inconclusive and warrant further high-quality, adequately powered trials.

The most consistent benefits were observed with interventions targeting the left DLPFC through a-tDCS, either alone or combined with cognitive tasks [39,41,45,46]. The use of anodal stimulation at intensities around 2 mA for approximately 20 min is consistent with protocols known to increase cortical excitability and facilitate long-term potentiation-like plasticity mechanisms. These improvements were typically measured in tasks sensitive to EFs, such as the SCWT, the CTMT2, or digit span assessments. These results are compatible with the established role of the DLPFC in regulating top-down control processes [28,57,58] and the growing evidence that neurostimulation may enhance neuroplasticity in populations with chronic pain [59,60]. Beyond focal alterations in the dorsolateral prefrontal cortex, chronic pain has been increasingly conceptualized as a disorder involving large-scale network dysregulation [61,62]. Neuroimaging studies have demonstrated altered functional connectivity within and between the frontoparietal control network, the salience network, and the default mode network in individuals with persistent pain [63]. Such network-level disruptions may compromise top-down regulatory processes, attentional control, and cognitive flexibility [64], thereby contributing to executive dysfunction. From this perspective, both neuromodulatory and exercise-based interventions may exert their EF effects not solely through regional cortical excitability changes, but also via modulation of distributed prefrontal-centered networks implicated in cognitive control.

However, the included studies exhibited substantial heterogeneity, which hinders the comparability of findings across trials and may explain some of the inconsistencies observed.

Several studies employed combined interventions. While such multi-modal approaches may reflect clinical practice more closely, they complicate the attribution of effects to individual components. As such, the specific contribution of physiotherapy-based strategies to EF improvement remains uncertain in these contexts, and future studies should include factorial designs or dismantling approaches to clarify active ingredients.

Beyond neuromodulation, a subset of studies suggested beneficial effects of physical exercise, both aerobic and sensorimotor-based, on EFs, such as working memory and attention [30,65,66,67]. Notably, aerobic-based interventions (e.g., treadmill and aquatic exercise) and rhythm-based activities such as Zumba were associated with improvements in working memory and attentional control [44,48]. These interventions may have required sustained cognitive engagement, motor coordination, and adaptation to dynamic stimuli, which may indirectly stimulate executive networks. The combination of motor planning, error monitoring, and attentional shifting inherent to these exercise modalities may partially explain the observed EF benefits. From a methodological standpoint, the exercise-based trials exhibited considerable variability in intervention design and prescription parameters. The frequency of training ranged from a single acute session (30–45 min) to programs delivered over 10, 12, or 16 weeks, with some protocols specifying three sessions per week. This heterogeneity in frequency, intensity, duration, type of exercise, and comparator design limits methodological comparability and hinders the identification of standardized exercise parameters associated with executive outcomes. The recent trial by Massah et al. [40] adds further support to this line of evidence, reporting short-term improvements in inhibitory control and cognitive flexibility following a structured exercise program. However, the study was judged to be at high risk of bias, and its findings should therefore be interpreted with caution.

It has been proposed that improvements in cognitive functions may occur through physiological mechanisms such as increased cerebral perfusion [68,69], although current literature has not established a direct link between perfusion changes and cognitive gains [24], along with the upregulation of neurotrophic factors (e.g., BDNF) [68,70,71,72], reductions in systemic inflammation [68,73,74], and other related pathways. Although these pathways are biologically plausible, current evidence remains indirect.

Notably, the potential of purely educational or behavioral physiotherapy interventions on executive functioning has been largely underrepresented in the current literature about EF outcomes. While some included studies combined education with neuromodulation, the isolated effects of these interventions remain underexplored. This represents an important research gap, especially considering that educational strategies aim to alter pain-related beliefs, attentional biases, and cognitive-emotional processing [75], all of which are linked to EFs [16,76,77].

Similarly, although physical exercise is widely recommended and frequently integrated into chronic pain management [78,79], its cognitive benefits may not be fully captured in trials that lack robust assessments. Future studies should adopt more comprehensive and validated cognitive batteries, report domain-specific effects, and consider long-term trajectories of EFs following exercise-based rehabilitation.

Taken together, these findings highlight the potential value of including EF outcomes as exploratory targets in chronic pain rehabilitation. Addressing these deficits may potentially contribute to improvements in quality of life, treatment adherence, self-regulation, and functional outcomes, although direct evidence supporting these downstream effects remains limited [80,81,82]. Therefore, expanding the scope to explore the EF impact of non-invasive, low-cost interventions such as exercise or educational strategies appears scientifically justified, although their clinical relevance for executive enhancement in chronic pain populations remains to be firmly established. An attempt in this direction has recently been made with the development of the pain-oriented biobehavioral therapeutic education approach, which explicitly incorporates cognitive, affective, and behavioral domains into patient education [83].

Future investigations should prioritize methodological rigor, larger sample sizes, and standardized EF outcomes. There is also a need to explore patient-level moderators (e.g., baseline cognitive performance, pain chronicity, psychological comorbidities) to better tailor interventions to individuals most likely to benefit from cognitive enhancement.

## 5. Limitations

This systematic review has several limitations that should be considered when interpreting the findings.

First, although only RCTs were included, many trials had relatively small sample sizes, potentially limiting statistical power to detect significant effects on executive functions.

Second, substantial heterogeneity was observed across studies in terms of intervention type, dosage, duration, comparator conditions, and outcome measures. Executive functions were assessed using diverse instruments, ranging from domain-specific neuropsychological tests to broader screening tools not specifically designed to isolate executive processes. This variability limits comparability across trials and precludes formal meta-analysis.

Third, several studies employed multimodal interventions combining physiotherapy with neuromodulation, education, or cognitive training, complicating attribution of effects to individual components.

Fourth, follow-up periods were generally short or absent, restricting conclusions regarding the long-term sustainability of EF improvements in a condition characterized by chronicity.

Fifth, the overall certainty of evidence was low to very low for most outcomes, reflecting methodological limitations, imprecision, and heterogeneity.

Sixth, grey literature was not systematically searched, which may increase the risk of publication bias, particularly in an emerging field where positive findings may be preferentially published.

Seventh, despite the randomized design, reporting quality varied, with insufficient detail in some trials regarding allocation concealment, assessor blinding, and intervention fidelity, introducing potential risk of bias.

Finally, the included populations were relatively homogeneous, with most studies conducted in middle-aged women with fibromyalgia, limiting generalizability to other chronic pain conditions and demographic groups.

Despite these limitations, this review provides a focused and methodologically transparent synthesis of the available evidence and identifies critical gaps for future research on executive function outcomes in physiotherapy-based chronic pain management.

## 6. Implications for Clinical Practice and Future Research

### 6.1. Clinical Implications

The findings of this review suggest that certain physiotherapy interventions may be associated with improving EFs in adults with chronic pain, with potential benefits for cognitive flexibility, attention, inhibitory control, and working memory. However, the current evidence remains preliminary, heterogeneous, and largely derived from small trials, and should therefore be interpreted as exploratory rather than confirmatory. These EFs are critically involved in pain modulation, emotional regulation, treatment adherence, and decision-making functions that are often impaired in chronic pain individuals.

From a clinical standpoint, the inclusion of these outcomes in physiotherapeutic interventions may contribute to a more comprehensive understanding of patient functioning, rather than representing an important step toward more holistic and patient-centered pain management. Neuromodulation techniques, particularly when combined with cognitive stimulation or education, have demonstrated preliminary efficacy and should not yet be considered established adjunctive therapeutic tools, given the limited and inconsistent evidence currently available.

Similarly, structured physical activity programs have shown signals of potential benefit in selected contexts. These interventions are low-cost, accessible, and may offer a feasible framework for simultaneously addressing physical and cognitive dimensions of chronic pain. However, current evidence is insufficient to support standardized protocols specifically targeting executive function enhancement within physiotherapy practice. Given their feasibility and potential benefits, they should be interpreted as promising but not yet definitive strategies for integrating EF outcomes into routine physiotherapy-based pain management.

However, clinicians should exercise caution when generalizing these findings, as not all interventions demonstrated consistent benefits, and individual responses may vary. In addition, differences in intervention type, intensity, and duration, as well as variability in outcome measures and follow-up periods across trials, further limit the ability to draw firm conclusions. These considerations highlight the importance of tailoring interventions to patient characteristics, closely monitoring EF as well as physical outcomes, and integrating physiotherapy strategies within a broader multidisciplinary framework that acknowledges the heterogeneity of chronic pain populations. Future adequately powered trials with standardized executive outcomes and longer follow-up periods are required before firm clinical recommendations can be formulated.

### 6.2. Research Implications

The results of this review also highlight several important directions for future research. First, there is a clear need for larger, high-quality RCTs with adequate sample sizes, standardized outcome measures, and long-term follow-up to better determine the magnitude and durability of EF changes.

Second, future studies should assess EFs as a primary or co-primary outcome, rather than as a secondary measure. This would improve the specificity and interpretability of findings.

Third, educational and behavioral physiotherapy approaches remain underexplored in terms of their EF effects. Given their focus on cognitive-emotional processing and pain beliefs, these interventions could plausibly influence EFs and warrant targeted investigation.

Finally, multimodal intervention studies that combine physical, cognitive, and neuro-modulatory components may offer synergistic effects and represent a promising avenue for integrative chronic pain rehabilitation.

## 7. Conclusions

Preliminary findings suggest that structured exercise and neuromodulation have potential for improving EFs (attention, working memory, and inhibitory control) in patients with chronic pain. However, the certainty of current evidence is low to very low due to methodological limitations, small sample sizes, and marked heterogeneity in interventions and outcome measures. Consequently, strong clinical recommendations cannot yet be made. Interventions combining physical and cognitive engagement appear particularly promising but require further investigation to isolate specific effects. Future research must prioritize rigorous RCTs with standardized definitions of executive function and longitudinal follow-up to determine if short-term cognitive gains translate into meaningful functional benefits for this population.

## Figures and Tables

**Table 2:** Methodological quality of the included RCTs assessed with the PEDro scale. Each item is reported as Yes (criterion satisfied) or No (criterion not satisfied). The column “Sum” indicates the total PEDro score, calculated as the sum of all positively rated items, from item 2 to item 11 (maximum score = 10). Higher scores reflect greater methodological quality.

No	Quality Assessment Scale	Alcon C et al. [39]	Vicuña Serrano P et al. [41]	Bilir I et al. [42]	Wu Y et al. [43]	Norouzi E et al. [44]	Souza Dos Santos V et al. [45]	Ferreira Silva A et al. [46]	Jay K et al. [47]	Munguía-Izquierdo D et al. [48]	Massah N et al. [40]
1	Eligibility criteria were specified	Yes	Yes	Yes	Yes	Yes	Yes	Yes	Yes	Yes	Yes
2	Subjects were randomly allocated to groups	Yes	Yes	Yes	Yes	Yes	Yes	Yes	Yes	Yes	Yes
3	Allocation was concealed	Yes	Yes	Yes	Yes	No	Yes	Yes	Yes	No	Yes
4	The groups were similar at baseline regarding the most important prognostic indicators	Yes	Yes	Yes	Yes	Yes	Yes	Yes	Yes	Yes	Yes
5	There was a blinding of all subjects	Yes	Yes	Yes	Yes	No	Yes	Yes	No	No	Yes
6	There was blinding of all therapists who administered the therapy	Yes	Yes	Yes	No	No	Yes	Yes	No	No	No
7	There was blinding of all assessors who measured at least one key outcome	Yes	Yes	Yes	Yes	No	Yes	Yes	Yes	Yes	No
8	Measures of at least one key were obtained from more than 85% of the subjects initially allocated to groups	Yes	Yes	Yes	Yes	Yes	Yes	Yes	Yes	Yes	Yes
9	Intention to treat	No	Yes	No	Yes	No	No	No	Yes	Yes	Yes
10	The results of between group statistics comparisons are reported for at least one key outcome	Yes	Yes	Yes	Yes	Yes	Yes	Yes	Yes	Yes	Yes
11	The study provides both point measures and measures of variability for at least one key outcome	Yes	Yes	Yes	Yes	Yes	Yes	Yes	Yes	Yes	Yes
	Sum	9	10	9	9	5	9	9	8	7	8

**Table 3:** Executive function domains were defined according to established neuropsychological models [18]. When a test involved multiple executive components, classification reflects the primary cognitive demand most consistently described in the literature.

Neuropsychological Test	Primary Executive Function Domain
SCWT [49]	Inhibitory control
CTMT [50,51]	Cognitive flexibility
Trail Making Test—Part B [49]	Cognitive flexibility/Executive control
WCST [49]	Cognitive flexibility
PASAT [52]	Working memory
Verbal Fluency Test—Semantic [49]	Executive retrieval/Cognitive flexibility
Verbal Fluency Test—Phonemic (FAS) [49]	Inhibitory control/Executive retrieval

## Data Availability

The original contributions presented in this study are included in the article/Supplementary Material. Further inquiries can be directed to the corresponding authors.

## Supplementary Materials

The following supporting information can be downloaded at https://www.mdpi.com/article/10.3390/neurolint18030055/s1, Supplementary File S1: PRISMA checklist.

## Abbreviations

The following abbreviations are used in this manuscript:

EFs	Executive Functions
RCTs	Randomized controlled trials
PRISMA	Preferred Reporting Items for Systematic Reviews
MeSH	Medical Subject Headings
RoB 2	Revised Cochrane Risk of Bias Tool for Randomized Trials
PEDro	Physiotherapy Evidence Databas
tDCS	Transcranial direct current stimulation
PCMT	Physical Cognitive Mindfulness training
rTMS	Transcranial magnetic stimulation
ICC	Intraclass correlation coefficient
DLPFC	Dorsolateral prefrontal cortex
PNE	Pain neuroscience education
SCWT	Stroop color word test
CTMT2	Comprehensive trail making test
a-tDCS	Anodal Transcranial direct current stimulation
PASAT	Paced auditory serial addition task
PCMT	Physical-cognitive-mindfulness training
WCST	Wisconsin card sorting test

## Appendix A. Complete Search Strategies for Each Database

Full search strings used in each database (PubMed, Scopus, Embase, CINAHL, and PsycInfo), including Boolean operators, MeSH terms, and syntax adapted to each platform. Searches were conducted in December 2024 and July 2025.

Search Strategy for MEDLINE:

(“Chronic Pain”[MeSH] OR “Persistent Pain” OR “Chronic Musculoskeletal Pain” OR “Musculoskeletal Pain”[MeSH] OR “Chronic Primary Pain” OR “Chronic Secondary Pain” OR “Chronic Widespread Pain” OR “Fibromyalgia” OR “Neuropathic Pain”) AND (“Physical Therapy Modalities”[MeSH] OR “Physical Therapy” OR “Physiotherapy” OR “Rehabilitation” OR “Manual Therapy” OR “Exercise Therapy” OR “Therapeutic Exercise” OR “Therapeutic Education” OR “Patient Education” OR “Pain Education” OR “Cognitive Behavioral Therapy” OR “Mindfulness” OR “Motor Control Exercise” OR “Neuromuscular Re education” OR “Graded Activity” OR “Graded Exposure” OR “Functional Training” OR “Postural Training” OR “Strength Training” OR “Aerobic Exercise” OR “Proprioceptive Training” OR “Balance Training”) AND (“Executive Function” OR “Cognitive Function” OR “Cognitive Performance” OR “Problem Solving” OR “Attention” OR “Cognition” OR “Pattern Recognition” OR “Decision Making” OR “Reaction Time” OR “Processing Capacity” OR “Processing Speed” OR “Psychomotor Functions” OR “Psychomotor Performance” OR “Memory” OR “Cognitive Impairment” OR “Cognitive Function”)

Search Strategy for EMBASE (Elsevier):

(‘Chronic Pain’/exp OR ‘Persistent Pain’ OR ‘Chronic Musculoskeletal Pain’ OR ‘Musculoskeletal Pain’/exp OR ‘Chronic Primary Pain’ OR ‘Chronic Secondary Pain’ OR ‘Chronic Widespread Pain’ OR Fibromyalgia OR ‘Neuropathic Pain’) AND (‘Physical Therapy Modalities’/exp OR ‘Physical Therapy’ OR Physiotherapy OR Rehabilitation OR ‘Manual Therapy’ OR ‘Exercise Therapy’ OR ‘Therapeutic Exercise’ OR ‘Therapeutic Education’ OR ‘Patient Education’ OR ‘Pain Education’ OR ‘Cognitive Behavioral Therapy’ OR Mindfulness OR ‘Motor Control Exercise’ OR ‘Neuromuscular Re-education’ OR ‘Graded Activity’ OR ‘Graded Exposure’ OR ‘Functional Training’ OR ‘Postural Training’ OR ‘Strength Training’ OR ‘Aerobic Exercise’ OR ‘Proprioceptive Training’ OR ‘Balance Training’) AND (‘Executive Function’ OR ‘Cognitive Function’ OR ‘Cognitive Performance’ OR ‘Problem Solving’ OR Attention OR Cognition OR ‘Pattern Recognition’ OR ‘Decision Making’ OR ‘Reaction Time’ OR ‘Processing Capacity’ OR ‘Processing Speed’ OR ‘Psychomotor Functions’ OR ‘Psychomotor Performance’ OR Memory OR ‘Cognitive Impairment’ OR ‘Cognitive Function’)

Search Strategy for EBSCO (CINAHL)—Health Sciences Databases:

((MH “Chronic Pain+”) OR “Persistent Pain” OR “Chronic Musculoskeletal Pain” OR (MH “Musculoskeletal Pain+”) OR “Chronic Primary Pain” OR “Chronic Secondary Pain” OR “Chronic Widespread Pain” OR Fibromyalgia OR “Neuropathic Pain”) AND ((MH “Physical Therapy Modalities+”) OR “Physical Therapy” OR Physiotherapy OR Rehabilitation OR “Manual Therapy” OR “Exercise Therapy” OR “Therapeutic Exercise” OR “Therapeutic Education” OR “Patient Education” OR “Pain Education” OR “Cognitive Behavioral Therapy” OR Mindfulness OR “Motor Control Exercise” OR “Neuromuscular Re-education” OR “Graded Activity” OR “Graded Exposure” OR “Functional Training” OR “Postural Training” OR “Strength Training” OR “Aerobic Exercise” OR “Proprioceptive Training” OR “Balance Training”) AND (“Executive Function” OR “Cognitive Function” OR “Cognitive Performance” OR “Problem Solving” OR Attention OR Cognition OR “Pattern Recognition” OR “Decision Making” OR “Reaction Time” OR “Processing Capacity” OR “Processing Speed” OR “Psychomotor Functions” OR “Psychomotor Performance” OR Memory OR “Cognitive Impairment” OR “Cognitive Function”)

Search Strategy for SCOPUS:

(“Chronic Pain” OR “Persistent Pain” OR “Chronic Musculoskeletal Pain” OR “Musculoskeletal Pain” OR “Chronic Primary Pain” OR “Chronic Secondary Pain” OR “Chronic Widespread Pain” OR Fibromyalgia OR “Neuropathic Pain”) AND (“Physical Therapy Modalities” OR “Physical Therapy” OR Physiotherapy OR Rehabilitation OR “Manual Therapy” OR “Exercise Therapy” OR “Therapeutic Exercise” OR “Therapeutic Education” OR “Patient Education” OR “Pain Education” OR “Cognitive Behavioral Therapy” OR Mindfulness OR “Motor Control Exercise” OR “Neuromuscular Re-education” OR “Graded Activity” OR “Graded Exposure” OR “Functional Training” OR “Postural Training” OR “Strength Training” OR “Aerobic Exercise” OR “Proprioceptive Training” OR “Balance Training”) AND (“Executive Function” OR “Cognitive Function” OR “Cognitive Performance” OR “Problem Solving” OR Attention OR Cognition OR “Pattern Recognition” OR “Decision Making” OR “Reaction Time” OR “Processing Capacity” OR “Processing Speed” OR “Psychomotor Functions” OR “Psychomotor Performance” OR Memory OR “Cognitive Impairment” OR “Cognitive Function”)

Search Strategy for PsycInfo

(exp “Chronic Pain”/OR “Persistent Pain” OR “Chronic Musculoskeletal Pain” OR exp “Musculoskeletal Pain”/OR “Chronic Primary Pain” OR “Chronic Secondary Pain” OR “Chronic Widespread Pain” OR Fibromyalgia OR “Neuropathic Pain”) AND (exp “Physical Therapy Modalities”/OR “Physical Therapy” OR Physiotherapy OR Rehabilitation OR “Manual Therapy” OR “Exercise Therapy” OR “Therapeutic Exercise” OR “Therapeutic Education” OR “Patient Education” OR “Pain Education” OR “Cognitive Behavioral Therapy” OR Mindfulness OR “Motor Control Exercise” OR “Neuromuscular Re-education” OR “Graded Activity” OR “Graded Exposure” OR “Functional Training” OR “Postural Training” OR “Strength Training” OR “Aerobic Exercise” OR “Proprioceptive Training” OR “Balance Training”) AND (“Executive Function” OR “Cognitive Function” OR “Cognitive Performance” OR “Problem Solving” OR Attention OR Cognition OR “Pattern Recognition” OR “Decision Making” OR “Reaction Time” OR “Processing Capacity” OR “Processing Speed” OR “Psychomotor Functions” OR “Psychomotor Performance” OR Memory OR “Cognitive Impairment” OR “Cognitive Function”)
